# Paternal mitochondria from an *rmd-2, rmd-3, rmd-6* triple mutant are properly positioned in the *C. elegans* zygote

**DOI:** 10.17912/micropub.biology.000422

**Published:** 2021-07-19

**Authors:** Iris Y Juanico, Christina M Meyer, John E McCarthy, Ting Gong, Francis J McNally

**Affiliations:** 1 Dept. of Molecular and Cellular Biology, University of California, Davis, Davis, CA USA

## Abstract

RMD-1,2,3,6 (regulator of microtubule dynamics) is a family of homologous proteins conserved between humans and *C. elegans*. Human RMD-3/PTPIP51 is a mitochondrial protein that tethers mitochondria to the endoplasmic reticulum. *C. elegans* RMD-2, 3, and 6 are expressed in sperm. To test whether paternal RMD-2, 3, 6 might redundantly tether paternal mitochondria to maternal ER at fertilization, we generated an *rmd-2, rmd-3, rmd-6* triple mutant. Paternal mitochondria derived from control or triple mutant worms were concentrated in a cloud around the paternal DNA at the future posterior end of zygotes during meiosis. No significant difference was detected in the position of paternal mitochondria within the zygote nor in the position of paternal mitochondria relative to paternal DNA within the zygote. There was also no reduction in progeny viability between control and triple mutant worms.

**Figure 1.**
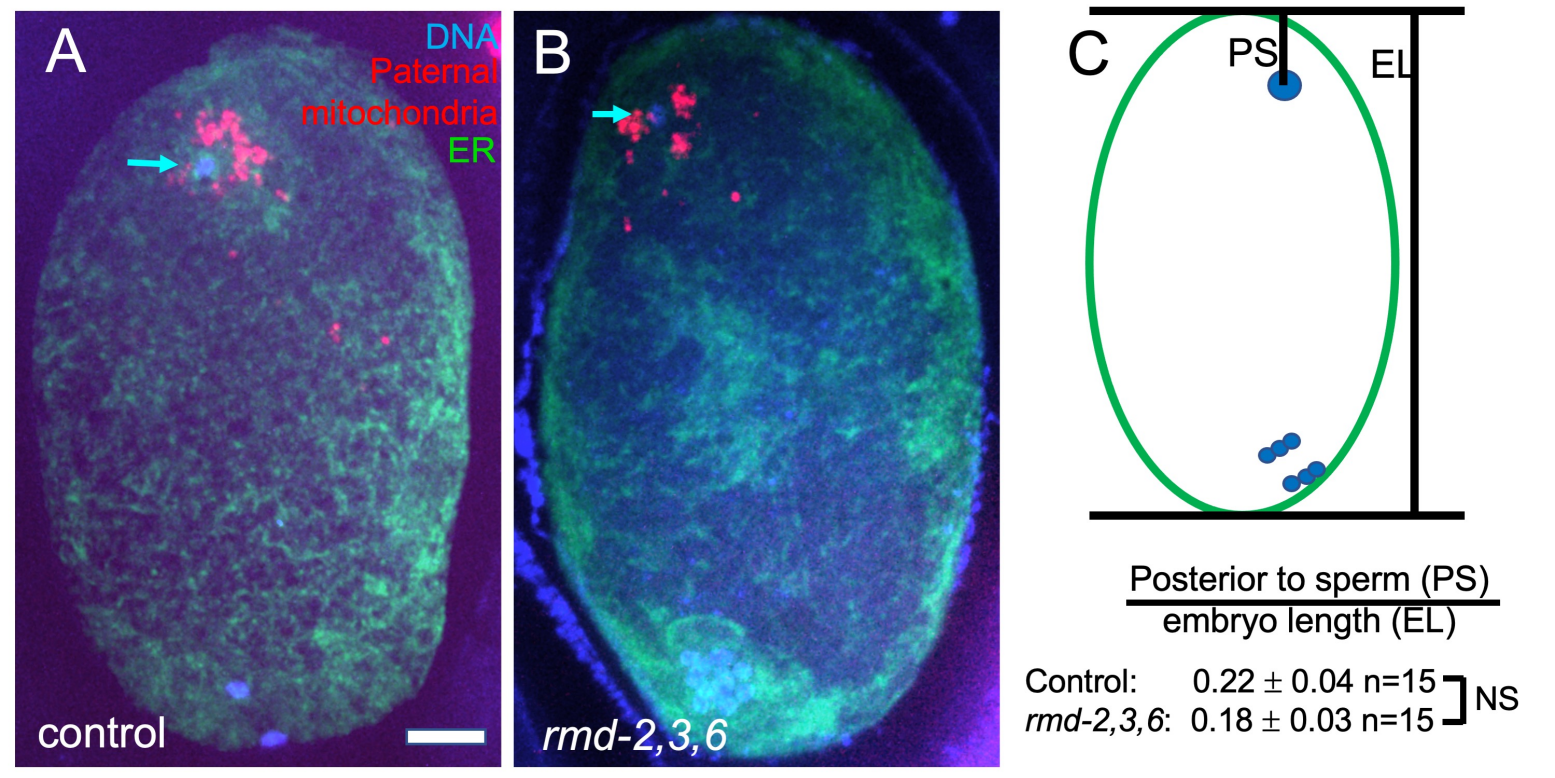
Position of paternal DNA within meiotic embryos. Hermaphrodites expressing GFP::SPCS-1, which labels the endoplasmic reticulum, were mated with control or *rmd-2, 3, 6* triple mutant males expressing MEV-1::mCherry, which labels mitochondria. (A, B) Representative z projections of methanol-fixed meiotic embryos stained with DAPI and anti-GFP antibodies. Blue indicates DAPI-stained DNA. Red indicates mCherry-labeled paternal mitochondria from the sperm. Green indicates maternal endoplasmic reticulum labeled with anti-GFP, which labels GFP::SPCS-1. Arrows indicate paternal DNA. Control and *rmd-2,3,6* indicate paternal genotypes. The control embryo shown is in anaphase I. The triple mutant embryo shown is in metaphase I. Bar = 5 um. (C) Diagram indicates how sperm DNA position relative to the future posterior tip was measured. PS indicates the distance from the future posterior tip of the ellipsoid embryo to the sperm DNA. EL indicates embryo length from the future posterior tip to the future anterior tip of the ellipsoid embryo. PS was divided by EL and the mean and SEM for the resulting ratios are shown. n indicates the number of embryos analyzed. Results of Students unpaired t test indicated no significant difference (NS) between control and *rmd-2,3,6* (p=0.42).

## Description

RMD-1 was discovered as a cytoplasmic regulator of microtubule dynamics in *C. elegans* and several highly homologous proteins were inferred from the *C. elegans* and human genome sequences (Oishi *et al.*, 2007). One of the human homologs, RMD-3/PTPIP51, has an N-terminal extension relative to other RMDs that targets it to the mitochondrial outer membrane and which binds the endoplasmic reticulum (ER) protein VAPB (De Vos *et al.*, 2012). This interaction mediates tethering of mitochondria to the ER in human cells (Stoica *et al.*, 2014). None of the *C. elegans* RMDs have sequence homology with the N-terminal extension found on human RMD-3/PTPIP51, however, *C. elegans* RMD-2 has an N-terminal extension relative to the other RMDs and this extension has potential to form a transmembrane helix as determined by TMpred release 25 (https://embnet.vital-it.ch/software/TMPRED_form.html). RMD-2 is present in sperm whereas RMD-3 and RMD-6 are highly enriched in sperm (Ma *et al.*, 2014). Paternal mitochondria are eventually degraded in the embryo (Sato and Sato, 2011). However, they are maintained, along with other sperm contents, at the opposite end of the zygote from the female meiotic spindle during meiosis (Fig. 1). We hypothesized that RMD-2, 3 and 6 on paternal mitochondria bind to the VAPB homolog, VPR-1, on maternal ER at fertilization to tether the sperm contents at the future posterior end of the zygote.

To test this hypothesis, we mated *rmd-2, rmd-3, rmd-6* triple mutant males bearing a transgene labeling mitochondria, *mCherry::mev-1* also called *mCherry::sdhc-1* (Trewin *et al.*, 2019), to hermaphrodites expressing a fluorescent marker of the ER. Resulting meiotic zygotes were fixed, stained with DAPI, and imaged (Fig. 1A-B). The position of the sperm DNA within the zygote was quantified (Fig. 1C) and no significant difference from control matings was observed. No qualitative difference was observed in distance of paternal mitochondria from the sperm DNA (Fig. 1A, B). Self progeny of triple mutant worms had a hatch rate that was not significantly less than progeny from control worms at 20°C (control KWN724: 94.9% hatching n=6 mothers, 731 progeny; triple deletion FM697: 97.6% hatching n=6 mothers 937 progeny).

Possible explanations are that RMD-1 carries out all the essential functions of this gene family, or that the triple mutant strain has a phenotype that was not assayed.

## Methods

*rmd-2(du8)* was generated by co-injecting KWN724 with two Cas9 RNPs (Upstream Guide Sequence + PAM (rev): tcagatgtaaagggtgacat cgg Downstream Guide Sequence + PAM (for): GCCACCAACTGCCACTGTCG AGG and the plasmid repair template: aaatcagaatgataagattaaaaatctccacaatcataaattgaaaagataagatagatctctataaataatgacgtcaactcttgcAATatgtcatct GCCACTGTCGAATAGGCTATCGCCGACTTCCAAGCAGCTCATGAAATCAATCCAGACTGGATTGAAAACACGGTTTACCTCGGAAAAGCTCTTCA using the *dpy-10* co-CRISPR method (Paix *et al.*, 2015). An *rmd-2* deletion was identified by PCR screening non-Dpy siblings of Dpy progeny. *rmd-2(du8)* removes exons 4, 5 and half of exon 6 of *rmd-2* isoform b. *rmd-6(du9)* was generated by co-injecting *rmd-2(du8)* worms with two Cas9 RNPs (Upstream Guide Sequence + PAM (rev): ATCGCGGTTCTTGGTTCCGA AGG Downstream Guide Sequence + PAM (rev): CGATGTCACACACTTCGGTA AGG and the ultramer repair template: ttaatcagtaccgatcaaccaccATGgtaagtttactccgatcttcaaatgtttaagctctttttcagAGTTTTGATGAAATTGACAAGAAATTCGGAAGCAAGTA taggtaactgaTGTGTGACATCGAACCGTATTCCGACGCTGAACAAGAGTTTGCCGATGATGCGAAGCAGATGTTGTCTAAGCTTTAAgttt using *dpy-10* co-CRISPR. PCR screening yielded an *rmd-6* deletion in a *dpy-10* worm. *rmd-6(du9)* removes all of exon 3 and half of exons 2 and 4. *rmd-3(tm2635)* was obtained from Shohei Mitani (National Bioresource Project for the Experimental Animal “Nematode C. elegans”). To make the triple mutant strain, *rmd-3(tm2635)* males were mated to *rmd-2(du8); rmd-6(du9); dpy-10; him-5; mev-1(jbm1 [mev-1::mCherry])* hermaphrodites. F2 self progeny of the F1 heterozygotes were allowed to lay eggs before being subjected to PCR screening for all three deletions. Some genotyping PCRs for *tm2635* yielded an apparent wild-type product, however, sequencing of this PCR product revealed it to be from *rmd-4*, which is annotated as a pseudogene.

Hatch rates were determined by singling L4 hermaphrodites, moving the worm to a fresh plate at 24 hr intervals, then counting eggs and larvae 48 hrs after removing adults from a plate.

## Reagents

**Table:** 

strain	genotype	available from
KWN724	*mev-1(jbm1 [mev-1::mCherry]) III him-5(e1490) V*	McNally lab
WH327	*unc-119(ed3) III; ojIs23 [pie-1p::GFP::spcs-1].*	CGC
FM697	*rmd-2(du8); rmd-3(tm2635); rmd-6(du9); mev-1(jbm1 [mev-1::mCherry]) III him-5(e1490) V*	McNally lab
